# Vortex Laser based on III-V semiconductor metasurface: direct generation of coherent Laguerre-Gauss modes carrying controlled orbital angular momentum

**DOI:** 10.1038/srep38156

**Published:** 2016-12-05

**Authors:** Mohamed S. Seghilani, Mikhael Myara, Mohamed Sellahi, Luc Legratiet, Isabelle Sagnes, Grégoire Beaudoin, Philippe Lalanne, Arnaud Garnache

**Affiliations:** 1 IES, CNRS-UMR 5214, Université Montpellier, France; 2 Laboratoire de Photonique et Nanostructures, CNRS-UPR 20, Marcoussis, France; 3 Laboratoire Photonique, Numérique et Nanosciences, CNRS-UMR5298, Institut d’Optique d’Aquitaine, Talence, France

## Abstract

The generation of a coherent state, supporting a large photon number, with controlled orbital-angular-momentum *L* = *ħl* (of charge *l* per photon) presents both fundamental and technological challenges: we demonstrate a surface-emitting laser, based on III-V semiconductor technology with an integrated metasurface, generating vortex-like coherent state in the Laguerre-Gauss basis. We use a first order phase perturbation to lift orbital degeneracy of wavefunctions, by introducing a weak anisotropy called here “orbital birefringence”, based on a dielectric metasurface. The azimuthal symmetry breakdown and non-linear laser dynamics create “orbital gain dichroism” allowing selecting vortex handedness. This coherent photonic device was characterized and studied, experimentally and theoretically. It exhibits a low divergence (<1°) diffraction limited beam, emitting 49 *mW* output power in the near-IR at *λ* ≃ 1 *μm*, a charge *l* = ±1, … ±4 (>50 *dB* vortex purity), and single frequency operation in a stable low noise regime (0.1% rms). Such high performance laser opens the path to widespread new photonic applications.

Optical vortex beams have known a growing interest since the first realization that they carry an Orbital Angular Momentum (OAM) 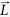
1 (Fig. 1). The development of such beams has led to many advanced applications such as optical handling of microscopic particles^2^, atoms manipulation^3^,^4^, sub-diffraction limit microscopy^5^, laser material processing^6^,^7^, and quantum information processing and telecommunication^8^,^9^,^10^,^11^,^12^.

Together with photon energy *ħω* and linear momentum *ħk*, the angular momentum 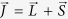 is one of the most important characteristics of light^1^. For paraxial fields in free space, the eigenmodes of 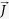 operator are circularly-polarized helically phased beams, where polarization helicity *σ* = ±1 (or 0 for linear state in anisotropic media) specifies the value of Spin Angular Momentum (SAM) per photon *S* = *σħ*, whereas the vortex integer topological charge *l* = 0, ±1, ±2, ... yields the OAM per photon *L* = *ħl*. The sign of *l* gives the direction of rotation of the wave-front. A striking difference between *L* and *S* momenta is the range of allowed values, while *σ* is bounded between −1 and +1, *l* can take much higher values. Vortex helical wave-fronts vary azimuthally with *θ* in a corkscrew-like manner along direction of propagation *z*, with a Poynting vector that follows a spiral trajectory around the axis. The wave-function reads as ∝*e*^*i*(*θl*−*kz*)^, with a phase structure containing *l* intertwined helices. The common transverse field profile looks like a light ring with a dark core and a phase singularity at the centre, known as doughnut-shaped modes (Fig. 1(a–d)).

Motivated by the potential of emerging applications, various methods have been used to generate OAM beams outside the laser cavity, such as spiral phase elements^13^, inhomogeneous birefringent plates (Q-plates)^14^ and holograms^15^, usually computer-generated using spatial light modulators. More recently, structured materials (also called metamaterials) have been used to generate and control the OAM of light^16^,^17^,^18^,^19^. This approach allows much more integrated devices and low loss especially when using all-dielectric metamaterials^20^,^21^.

Many works have been dedicated to generation and control of SAM inside a laser cavity, for example using electronic spin transfer for spintronic applications^22^,^23^. On the other side, generation of OAM inside a laser cavity is complex and comes with both technical and fundamental challenges. Nevertheless, it is a very promising approach to obtain high power, highly pure, coherent Laguerre-Gauss vortex mode and has been investigated through several experimental works^24^,^25^,^26^,^27^,^28^,^29^,^30^,^31^,^32^. Yet, a fundamental limitation for OAM handedness control arises from orbital degeneracy and symmetry of wave-functions with opposite orbital number ±*l* as in the hydrogen atom: in conventional optical cavities, the obtained OAM handedness is very often not controlled in a deterministic manner. Furthermore, in most of the reported works thick and movable elements are used inside the laser cavity, which limits the coherence and often the beam quality because of loss and misalignments, and results in cumbersome systems unsuitable for integration. Thus, many desired properties such as OAM purity, and power, come at the expense of other ones such as compactness, handedness control and coherence.

In this work we demonstrate for the first time to our knowledge, a compact III-V semiconductor surface-emitting laser with an integrated intra-cavity all-dielectric meta-surface^20^,^21^, that generates high power, highly coherent OAM modes in the Laguerre-Gauss basis with controlled OAM charge. For handedness control, we use a first order phase perturbation to lift degeneracy and to create optical “orbital anisotropy” for counter-rotating vortices. This allows to select the laser vortex handedness by exploiting H. Haken’s principle^33^ “Darwin’s survival of the fittest”.

## The problem of vortex handedness degeneracy and control: OAM beam generation

Optical vortex-beams are most commonly assumed to belong to Laguerre-Gaussian 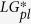 mode basis (or Bessel-Gaussian). The total intracavity field 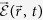 and the transverse LG spatial wave-functions Ψ_*qpl*_(*r, θ, z*) for a stable plano-concave-type optical cavity (free space, length *L*_*c*_, radius of curvature *R*_*c*_), reads as^34^:


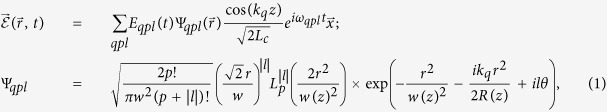


where *E*_*qpl*_(*t*) are slowly time varying envelopes and 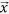 the polarization unit vector. *r, θ, z* are the radial, azimuthal and longitudinal coordinates. 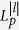 is the generalized Laguerre polynomial; *p (q*) is the radial (longitudinal) number (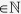); *R*(*z*) is the wavefront radius of curvature; *w*(*z*) ≫ *λ* is the Gaussian beam waist, *z*_*R*_ = *πw*^2^/*λ* is the Rayleigh length and *λ* is the wavelength. *ħω*_*qpl*_ are eigen-energies for *q, p, l* quantum numbers (Fig. 2(c)), given by the photon energy-momentum dispersion relation, assuming a unique polarization state^34^:


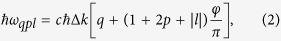


where 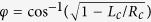 is the resonator Gouy phase shift; *ħ*Δ*k* = *ħ*2*πFSR*/*c* is the quantum of momentum; *FSR* = *c*/2*L*_*c*_ is the cavity free spectral range.

**Figure 1:**
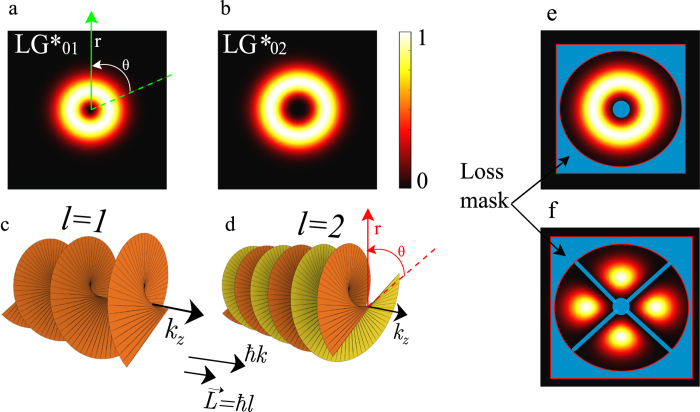
Transverse intensity profile of (**a**) 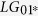 and (**b**) 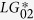 modes and their respective helical phase structures (**c**), (**d**). Transverse intensity profiles of (**e**) 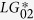 and (**f**) degenerate *LG*_02_ along with intracavity loss masks needed for their selection.

A fundamental advantage of using confined states (with *φ* ≠ 0) compared to continuum states, is the energy degeneracy lift for different quantum numbers *q, p*, |*l*|: they are distinguishable spatially and spectrally. However, the orbital degeneracy and symmetry for vortices of opposite quantum number ±*l* is clear from Eqs (1, 2): they can not be selected using an intracavity transverse intensity filter, and their linear combination *LG* is an eigenstate with *L* = 0 and 2*l* intensity lobes (called “degenerate” modes). It is possible to select a given *LG* or *LG*^∗^ mode of quantum number *p*, |*l*|, by distributing transverse optical losses (gain) in zero (maximum) intensity regions (see Fig. 1(e,f)). For this purpose, an intracavity metallic mask or patterned pump beam can be applied on the axis^26^,^27^,^35^. But introducing losses in the dark core and periphery region of a 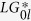 mode results in 50% probability of creating either right-handed or left-handed stable vortex wave, each time the laser builds-up^27^,^35^. Thus direct laser generation of vortex beam appears fundamentally challenging.

**Figure 2:**
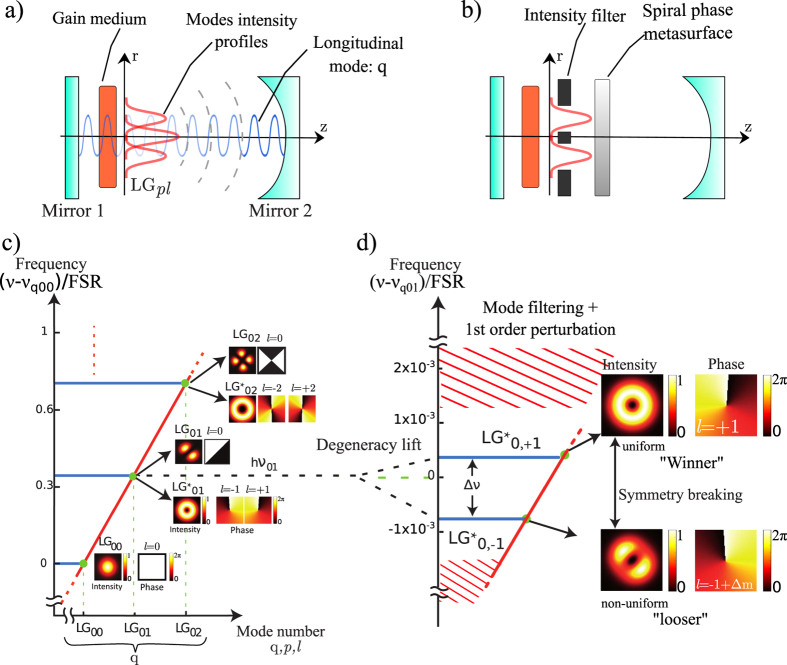
Schematics of (**a**) Plano-concave type cavity supporting LG basis, and (**b**) same cavity with an intensity selective filter and SPM. (**c**) Photon energy (frequency *ν*) -momentum (quantum number) dispersion diagram for the first three non degenerate 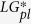 and degenerate *LG*_*pl*_ transverse modes, with longitudinal mode number *q*; modes having same OAM charge but opposite handedness have degenerate frequencies and symmetric wave-functions; momenta are normalized to *ħ*Δ*kφ* (see Eq. 2). (**d**) Introduction of the SPM lifts orbital frequency degeneracy and breaks orbital wave-function symmetry for 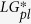 modes with opposite charge ±*l*.

Here we “break” this fundamental laser light symmetry problem in a deterministic way, thanks to a robust physical and technological concept (Fig. 2(b,d)), of great interest for demanding applications: we use a first order phase perturbation to lift degeneracy, by introducing a weak “orbital anisotropy” (“orbital birefringence” here), based on a dielectric meta-surface. The induced azimuthal symmetry breakdown creates gain anisotropy, called here “orbital gain dichroism”, arising during non-linear dynamics of semiconductor laser.

**Figure 3:**
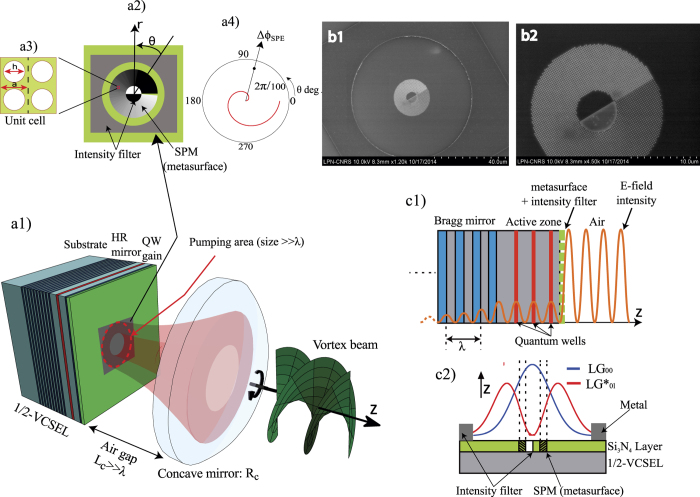
(**a1**) Schematic description of the vortex semiconductor-laser, based on III-V semiconductor technology. Stable cavity with a millimetre air gap *L*_*c*_. Pump diameter 90 *μm*. (**a2**) SPM and intensity filter (loss masks). (**a3**) Elementary cell of the 2D sub-wavelength grating forming the metasurface. (**a4**) Perturbative azimuthal phase distribution Δ*ϕ*_*SPM*_ induced by the SPM. (**b1**) Scanning electron microscope image of fabricated right-handed SPM surrounded by a Chromium metallic mask. (**b2**) Zoom on SPM (20 *μm* external diameter). (**c1**) Longitudinal E-field distribution in the 1/2 VCSEL structure and SPM position. (**c2**) Longitudinal (XZ) cross section showing the overlap of the *LG*_00_ and *LG*_01_ modes with the SPM and the intensity filter, the peak of unwanted *LG*_00_ mode coincides with the high central loss.

Our direct laser generation demonstration of *LG*^∗^ modes is achieved thanks to a high finesse compact vertical-external-cavity-surface-emitting-laser (VECSEL)^36^ based on III-V semiconductor technology emitting in the Near-IR at 1 *μ*m. The stable laser cavity exhibits a low noise, relaxation oscillation free (class-A) dynamics^33^,^36^,^37^ for high coherence. We used a sub-wavelength tick (*λ*/8) all dielectric metamaterial layer (metasurface) integrated onto a 1 *μm* thick Quantum-Wells (QW) based gain mirror structure^20^, called “1/2-VCSEL” (see Fig. 3(a1)). A few nanometers tick metallic layer is deposited on top of the metasurface, the combination of these two layers allow the right quantum numbers *p, l* to be selected under laser phase transition thanks to transverse spatial-hole-burning (SHB) that occurs in the gain medium^33^,^34^. This source generates a single - transverse (*p, l*), longitudinal *q*, and polarization *S* - coherent light state at moderate power.

## OAM’s charge and sign control in laser cavity

### Charge selection, degeneracy lift and symmetry breakdown of cavity eigenmodes

The purpose of this section is to build the cold cavity eigen-basis, in order to reduce to minimum the basis dimension (q, p, l), and create the only-desired *LG*^∗^ non-degenerate modes. We used first order perturbation theory like in quantum mechanics as a tool. The metasurface acts as a transverse loss filter and introduces weak “orbital birefringence” (Fig. 2(b,d)). Figure 3(a1) shows a schematic representation of our two mirror standing wave laser cavity with integrated metasurface on the 1/2 VCSEL.

Figure 3(a1 presents the three building blocks required for intracavity OAM generation and control: (i) an axially symmetric stable free-space plano-concave cavity^34^; (ii) a sub-wavelength thick selective transverse intensity filter; (iii) a Spiral Phase Metasurface (SPM).

The first building block, the cavity, has a finesse of 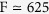. It operates far from degeneracy at low Fresnel number, in order to be able to select a transverse mode number (*p*,|*l*|) in a discrete *LG*^∗^ basis of size *w* ≫ *λ* here. It consists of a highly reflective gain mirror containing a distributed Bragg reflector on GaAs substrate, followed by a 1 *μm (λ*) thick multi-QW-based gain region ended by a window, a millimetre-long *L*_*c*_ air gap, and a dielectric concave mirror as output coupler. The non-degenerate eigen-frequencies *ν*_*qpl*_ = *ω*_*qpl*_/2*π* (Fig. 2(c)) are given by Eq. 2. Only azimuthal degeneracy and symmetry for *l* = ±*m* (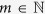) remain in this basis. It is important to note that the SPM and the intensity filter are located close to a node of the E-field vertical standing wave and on cavity end mirror: their effect is weak and homogeneous over all *q* modes within mirror bandwidth.

The intensity filter has the function of OAM charge control, i.e. the selection of a “doughnut” mode numbers (0, |*l*| = *m*). This function is basically obtained by transversely distributing large optical losses in the dark regions of the desired mode, namely the dark core and the peripheral region, this “kills” the finesse of undesired modes and prevents them from reaching lasing threshold. In our case, the high loss in the dark core is obtained using a central abrupt phase-step of about 2*π*/5 (diffraction loss) implemented by processing the subwavelength thick (*λ*/8) dielectric layer on the 1/2-VCSEL top surface: the dielectric material in half the central circular region is etched creating a refractive index contrast. The high loss in the outer peripheral region is implemented using a few nanometers-thick metallic mask^27^ longitudinally located close to field antinode, ensuring ultra-low diffraction loss for the vortex beam. Both the phase-step and the metallic mask forming the intensity filter are shown schematically in Fig. 3(a2), and in the micro-graphs of Fig. 3(b1) and (b2). Alternatively, a similar filtering of the outer region can be obtained thanks to finite gain -pumping- region diameter. This scheme allows to control the charge *m*, by changing the transverse dimensions of the intensity filter to fit with another mode with different waist and OAM charge. The same effect can be obtained by changing the cavity length while keeping the same intensity filter. Because as required by the cavity stability, the mode’s waist varies with the cavity length, thus by varying this latter, the OAM jumps as the LG mode waist changes.

The last building block, the SPM, has the function of OAM handedness control *l* = ±*m*. To do this, the idea is to weakly perturb the cavity *LG*^∗^ eigenbasis without breaking wave-function orthogonality, by introducing “orbital birefringence” acting as a first order asymmetric azimuthal perturbation (Fig. 2(b,d)). The SPM consists in a subwavelength (*λ*/8)-thick dielectric layer, perforated by a 2D array of holes (of diameter *h*) placed on a square grid, of period *a* (Fig. 3(a2)). This 2D sub-wavelength grating acts as a metamaterial with a refractive index equal to the normalized propagation constant of fundamental Bloch mode, controlled through the filling factor *h*/*a*^20^.

**Figure 4:**
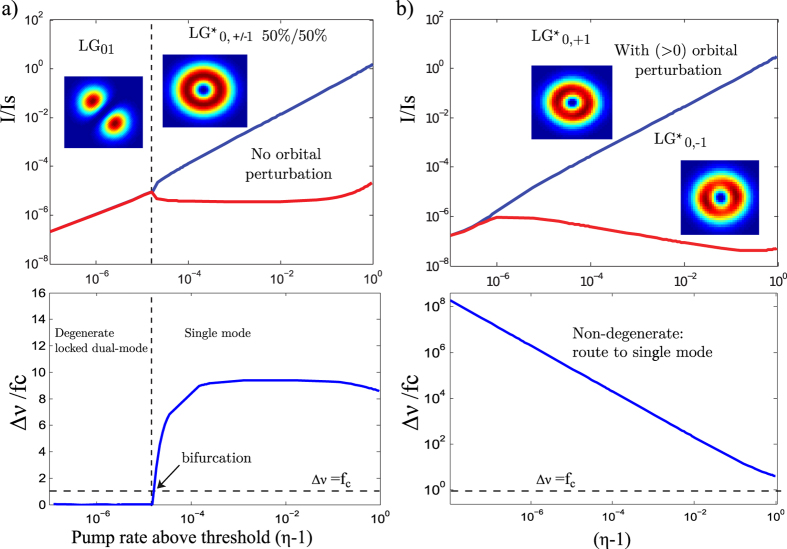
Upper plots: Steady state diagram of the dual-transverse mode laser intensities (normalized to saturation intensity *I*_*s*_) as a function of pumping rate *η* for two contra-rotating vortex modes. (**a**) Cavity without phase perturbation; here both handednesses have 50% probability to be generated. (**b**) With orbital phase perturbation (SPM): here handedness is deterministically controlled and only the wanted one is generated thanks to asymmetric mode competition. In (**b**), the transverse modes profiles were obtained using an in-house 2D eigenmode solver. Lower plots show the laser frequency degeneracy lift for case (**a**) and (**b**), normalized to the laser cavity frequency cutoff *f*_*c*_(*η*).

To fabricate the SPM, the hole diameter *h* is varyied with the angular coordinate *θ*, the function *h*(*θ*) is obtained in two steps: first the effective refractive is calculated as a function of the hole diameter *n*_*eff*_(*h*) using rigorous coupled-wave analysis method^20^,^38^, next the inverse function *h*(*n*_*eff*_) is used to calculate the hole diameter profile *h*(*θ*) required to implement the targeted refractive index (or equivalently the phase) profile *n*_*eff*_(*θ*)^20^. Here the targeted phase profile is a weak azimuthal phase variation with an amplitude Δ*ϕ*_*SPM*_ ≪ 2*π* for a complete turn. Estimated optimal value of Δ*ϕ*_*SPM*_ is


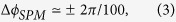


with details given below. Figure 3(a3) shows the fabricated SPM, azimuthal variation of hole diameter can be seen from the micrograph, details of fabrication are given in Methods section. The SPM is either located on dark core side or periphery of the vortex mode. This diffracting, non-reciprocal, azimuthal SPM will lift degeneracy and break orbital symmetry of contra-rotating eigenmodes: they thus become distinguishable. Therefore, in our cavity a vortex rotating in opposite direction to that of SPM (>0 for example), experiences slight OAM reduction *l* = −*m* + Δ*m*^39^ every round-trip : azimuthal symmetry is broken. This reduction is proportional to round trip modal phase shift Δ*ϕ*_*modal*_ brought by perturbation:


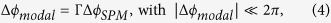


where Γ is the transverse overlap factor between the beam and the SPM, given by:


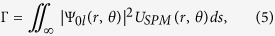


where *U*_*SPM*_ is the SPM surface distribution function on the 1/2-VCSEL. This way we end up with two new non-degenerate asymmetric wavefunctions given by a linear combination of *LG*^∗^ eigenmodes: the less perturbed one of charge 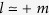 (>0 for example) conserves almost a homogeneous doughnut-like intensity distribution, while the opposite one of non-integer charge 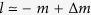 shows significant azimuthal intensity modulation (with 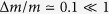 in case of Fig. 4b). As a result the phase of each contra-rotating mode will experience different intracavity Gouy-shift (Eq. 1)^34^. One should then expect a slight optical frequency degeneracy lift (positive for Δ*m* > 0) of amplitude 2*π*Δ*ν* = |*ω*_*q*,0,*m*_ − *ω*_*q*,0,−*m*_|:


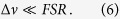


To ensure efficient laser dynamics unlocking of the two contra-rotating waves - even in the presence of azimuthal back scattering like in gyro-laser^33^,^34^, one should choose a modal phase perturbation strong enough,


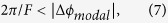


so that Δ*ν* is greater than the cold cavity frequency cutoff *f*_*cc*_,


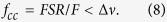


Thus, a high *F* releases the strain on Δ*ν* value to be in perturbative regime. It is worth noting that the degeneracy lift may result in several sub-states. The frequency splitting is predicted by our numerical calculations of the perturbed eigen-basis^40^,^41^ (see Materials and Methods sec. 2). It is also noteworthy that, under such weak spatial perturbation, the two contra-rotating waves still have negligible diffraction losses, allowing the existence of an orthogonal mode basis. In Fig. 3(b2) we show a scanning electron microscope photograph of the fabricated SPM with right handedness, and in Fig. 3(b1) we show the SPM surrounded by a metallic mask, as peripheral loss filter. The beam diameter is ≫*λ*, typically 100 *μm* here for low divergence and high power operation.

To end, the spin angular momentum is *S* = 0 here, because a III-V 1/2-VCSEL gain mirror exhibits weak linear birefringence, therefore the two non-degenerate polarization eigenstates are linear, oriented along [110] and [1–10] crystal axis^36^,^37^.

### Light-matter interaction in the gain medium and mode competition dynamics

Now comes the role of weak light-matter interaction in the gain medium and semi-classical non-linear laser dynamics to generate a single coherent light state *E*_*q*0*l*_ at large photon number during phase transition. The intracavity SPM design (for Δ*m* > 0) allows only four non-degenerate frequency combs *q* to oscillate on 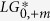 and 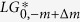 (slightly non integer charge) modes, for both [110] or [1–10] polarization axis.

First, thanks to homogeneous and (*r, θ*) quasi-isotropic QW gain mirror properties, emitting vertically along the quantization axis [001] - under interband transition between continuum electronic states -, the energy *ħω*, the linear momentum *ħk* and the angular momentum *J* can be transferred from a “macroscopic” electronic wavefunction to a single photon state wavefunction (of volume ≫*λ*^3^) through stimulated emission. This point has been recently theoretically demonstrated in ref. 42.

Secondly, one of the two OAM signs will be selected, thanks to non-linear dynamics during laser build-up and the well known transverse SHB effect of the electronic population^34^. This non-linear mode competition, called by H. Haken “Darwin’s survival of the fittest”^33^ always favour a laser state with an homogeneous intensity pattern, for efficient use of the gain under saturation. As shown in Fig. 4(a,c), this explains why when one tries to select a vortex laser state 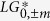 without lifting the degeneracy of contra-rotating eigenmodes, at pumping rate far enough above threshold (to prevent modelocking due back scattering like in gyrolaser^33^,^34^), one obtains 50% probability of having either right-handed or left-handed stable vortex mode each time the laser is switched on, due to purely random nature of spontaneous emission assisted seeding^27^,^35^. Indeed, the superposition of two oppositely rotating degenerate vortex modes will produce azimuthal standing wave with *L* = 0, leading to significant azimuthal SHB and unstable operation of two locked vortex modes due to larger cross saturation of the gain. In the case of asymmetrical degeneracy lift, the unwanted mode 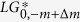 exhibits its own azimuthal standing wave pattern, leading to a larger and asymmetric self-saturation: it creates like an “orbital gain dichroism” favouring 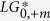.

Finally, here *S* = 0 and linear gain dichroism will strongly select a single linear polarization state along [110] crystal axis^36^,^37^. Moreover, thanks to subwavelength thick QW gain layer localized on longitudinal standing-wave antinode of all *q* modes (within the gain bandwidth), together with QW homogeneous broadening, a single mode *q* will be selected^37^.

The semi-classical non-linear dynamics between these two - degenerate or not - modes are modeled by a dual-mode spatio-temporal Maxwell-Bloch equation set^33^,^34^. Here the carrier relaxation rate *A* is fast compared to 2*πf*_*cc*_ leading to a class-A laser dynamics free of relaxation oscillations. We took account of SPM effect, self-saturation and cross saturation coefficients due to SHB, as well as spontaneous emission noise (Langevin forces) and back scattering (see Methods Sec. 2). Now we move to the laser phase diagram for both intensity and frequency of the E-field. Numerical simulation results of cavity eigenstates and vortex laser states competition are summarized in Fig. 4. The mode steady state intensities are plotted, together with the frequency degeneracy lift Δ*ν*, as a function of the normalized pumping rate above threshold *η*, for a dual-transverse mode laser (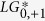 and 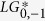). The frequency degeneracy lift is normalized to the laser cavity cut-off^34^:


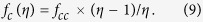


Two cases are simulated: a cavity without phase perturbation (no SPM) (Fig. 4(a,c)) and a cavity with phase perturbation (Fig. 4(b,d)). In the first case the two modes are degenerate and locked up to a certain pumping rate, after which a bifurcation occurs and only one mode survives. The choice here is purely random and set by quantum noise assisted seeding. In the second case thanks to degeneracy lift, locking is no more possible. The dual- to single-mode second threshold is defined by spontaneous emission, and is very low. More importantly handedness selection is deterministic. These results agree with the experiment addressed in the next section.

## Vortex Laser and physical study

**Figure 5:**
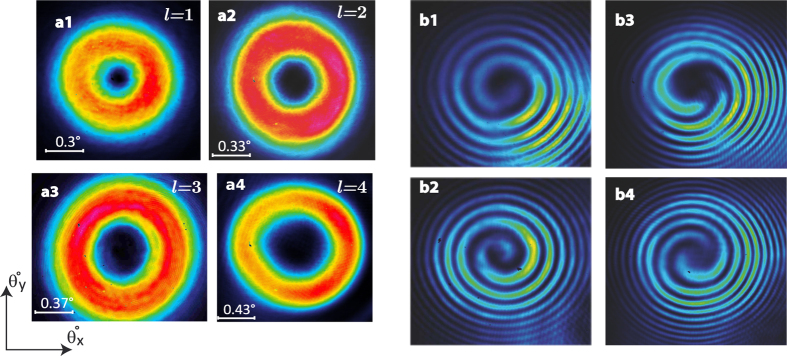
Intensity profiles of obtained LG^*^ modes having helical wavefront: (**a1**) 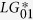, (**a2**) 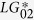, (**a3**) 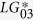 and (**a4**) 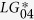. Mach-Zehnder Interferogram: (**b1** (**b2**)) One-start spiral fringes corresponding to right (left) handed 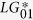 with OAM charge *l* = 1(−1). (**b3** (**b4**)) Two-starts spiral fringes corresponding to right (left) handed 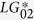 with OAM charge *l* = 2(−2).

The laser is emitting at 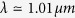, and exhibits a low threshold pump density (0.8 *kW*/*cm*^2^), a high differential efficiency of 27% and a maximum output power of 49 *mW* limited by pump power. Figure 5(a) shows the far field intensity profiles obtained with lasers operating in continuous-wave on vortex modes of different orders 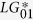 (a1), 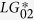 (a2), 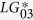 (a3), 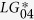 (a4). In the experiment, the charge |*l*| = 1, 2, 3... is changed using a single semiconductor chip by varying *L*_*c*_ over hundreds of microns. The handedness is changed by translating the pumped region to another SPM with opposite spiral phase handedness located on the same chip at 200 *μm* from the first one. In Fig. 5(b) we show corresponding spiral interference patterns of 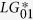 having right-(b1)/left-(b2) handedness, and 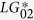 with right-(b3)/left-(b4) handedness. These patterns were obtained using interference of collimated vortex beam with a copy of itself having a curved wavefront in a Mach-Zehnder interferometer^43^. To achieve this scheme, a lens with a short focal length is inserted in one of the two arms of the interferometer, so as the beam is tightly focused before diverging rapidly. In addition to confirming the existence of the OAM, this interference technique allows its handedness to be determined from the fringes, while a conventional Mach-Zehnder interferometer would only confirm the existence of OAM. The obtained spiral fringes show a well-determined integer charge and handedness *l* proving the OAM control capability of the fabricated laser.

**Figure 6:**
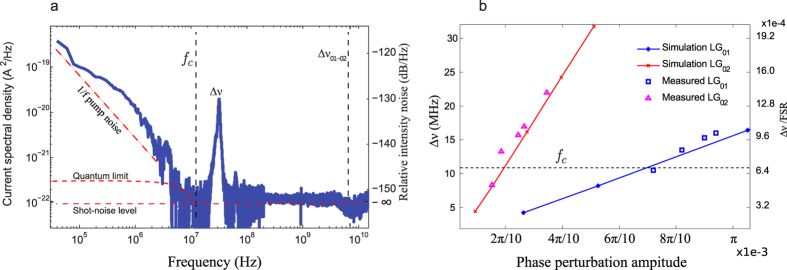
(**a**) Relative-Intensity-Noise RF spectrum of the vortex laser measured by integrating a fraction of the beam surface, for 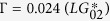 and *f*_*c*_ = 12 *MHz*. (**b**) Measured and simulated beat frequencies between selected 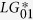 and 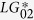 vortex modes and residual contra-rotating modes, as a function of the modal phase perturbation amplitude |Δ*ϕ*_*modal*_|. The cavity frequency cutoff *fc* = 12 *MHz (F* = 625, 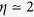) is represented by dashed horizontal line splitting the figure into two parts: upper and lower with strong and weak control of the OAM sign respectively.

The generated vortex beams exhibit high spatial coherence, with pure 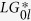 modes (see Fig. 6) close to diffraction limit. We measured a beam propagation parameter *M*^2^ of (1 + |*l*|) × 1.2(±0.2), where theoretical value reads *M*^2^ = (1 + 2*p* + |*l*|). Spiral fringes show that the generated beams possess a well defined single OAM and usually used in the literature as an indicator of its purity. However, this cannot be used to measure quantitatively the purity of the generated OAM. To evaluate the optical intensity suppression ratio *I*_−_/*I*_+_ of counter-rotating OAM, we measured the normalized beating power *P*_*e*_ between the two vortex fields at Δ*ν* in the laser RF power spectral density^34^ (see Methods Sec. 3).

Figure 6(a) plots the measured Relative-Intensity-Noise (RIN) of the vortex laser. It shows a low noise class-A laser dynamics - free of relaxation oscillations - with a cut-off frequency at *f*_*c*_ ≈ 12 *MHz*, and the expected weak beat note at 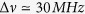 for 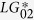. The measured opposite OAM suppression ratio is *I*_+_/*I*_−_ > 50 *dB* in this experiment. Then the RIN stays at the shot noise level until the next longitudinal mode of the cavity at *FSR* = 15.8 *GHz*. Dashed vertical line in Fig. 6 corresponds to first other order LG transverse mode beat frequency. However, in our case we have unique transverse mode with a good suppression ratio >50 *dB* as the RIN stays at shot noise level.

The degeneracy lift Δ*ν* is even stronger when the overlap Γ between the SPM and the mode is greater. This configuration will favour even higher opposite OAM suppression ratio at high pump rate by reducing residual coherent mode coupling (see Methods Sec. 3). We calculated Δ*ν* for different modal phase perturbation amplitude |Δ*ϕ*_*modal*_| and compared it with measured ones for 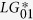 and 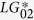 modes. Both values are in good agreement with the experiments, as shown in Fig. 6(b).

Finally, the coherent linear polarization state (*S* = 0) is along [110] crystal axis^37^, with an orthogonal polarization extinction ratio >60 *dB*, evaluated by measuring the cross-polarization optical beating power. Single frequency - longitudinal mode - operation with a side mode suppression ratio of 27 *dB* was obtained, showing a coherent vortex state.

## Discussion

The optical method demonstrated here shows an effective way to generate a pure coherent 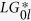 vortex mode exhibiting an integer charge value *l* = ±1, ±2, ... ±4. For this purpose, a low noise high finesse laser cavity integrating a meta-material based on III-V semiconductor flat-photonics technology has been developed. We used a first order orbital perturbation to lift azimuthal degeneracy and break spatial symmetry (called “orbital anisotropy”), in order to select the desired OAM during non-linear laser phase transition. This solution overcomes physical and technological limitations of conventional schemes in terms of vortex control, coherence and power. The big advantage of the approach presented here lies in: its physical and technological robustness; cavity design finesse, simplicity and symmetry (weak thermal lens, aberrations and astigmatism); no need for extra-intracavity optical elements; the subwavelength grating is fabricated using III-V nanotechnology exhibiting ultra low optical roughness and defect density, with a clear interest for the integration (industry-ready).

This generates a single frequency highly coherent and powerful (49 *mW*) low divergence (1°) diffraction-limited LG vortex beam, on a unique quantum number *q, p, l* and polarization state (high suppression ratio >30 *dB*). These beams are easy to manipulate and focused to spots <500 *nm* using standard commercial high NA optics. The vortex charge was here limited to *l* = 4 due to low alignment precision of standard mechanics (cavity astigmatism). Larger charge *l* ≫ 4 could be reached easily using high precision mechanics, until residual thermal lens astigmatism would limit cavity axial symmetry, backscattering would then lock the modes, or loss of rotational symmetry in light-matter interaction.

To explain the laser behaviour, we used the matrix method and the Fox-Li iterative technique to calculate the eigenmodes, as well as non-linear semi-classical Maxwell-Bloch dynamical equations to study the dual-vortex stability diagram of this class-A laser. In contrary to class-B laser dynamics (solid state lasers, monolithic VCSELs…), class-A dynamics (2*πfcc* ≪ *A, A* being carrier relaxation rate) allows to enforce vortex mode stability, avoiding petals like mode solution *l* = 0, due to efficient cross saturation with fast gain recovery in QW medium.

The purity and coherence of the vortex laser were quantified by measuring the RF spectrum of optical beat note with weak cavity eigenstates, close to shot noise above 10 *MHz* (class-A); this gives also a upper value for the fundamental Schawlow-Townes laser linewidth below 200 *kHz* (limited by quantum noise in the weak mode here). The theoretical fundamental vortex linewidth^36^ is about 1 Hz here (coherence time ~300 ms).

The vortex laser principle demonstrated here can be extended to high power above watt level, to any wavelengths and laser technology, as well to any temporal light state, as for example ultra-short soliton-like pulse operation at high repetition rate. In addition to the OAM, a spin angular momentum *S* = ±*ħ* control would be of great interest for spintronic applications or light spin-orbit manipulation.

Breaking the bottleneck of OAM’s charge and sign selection and control in a laser cavity is of crucial importance. Indeed, such high performances compact laser device opens the path to a new family of sensor of great interest for demanding photonic applications, such as optical nano-manipulation of biological particle, active micro-rheology, atom guiding and acceleration, manipulation of Bose-Einstein condensates. This would enable to achieve compact, high performances and cost-effective systems, as a vortex laser combined with self-mixing interferometry can be used at the same time as an optical tweezers and an auto-aligned, highly sensitive rotational and linear speed sensor.

The method demonstrated here is a static one, i.e one chip can select one OAM sign only: it can not be reversed dynamically; only the OAM charge |*l*| can be varied. However, the dynamic control of OAM sign can be realized here by varying cavity length *L*_*c*_ over ten’s of microns to change mode waist, thanks to a SPM design containing two contra-rotating SPM’s, one located inner and the other outer of vortex intensity distribution, to create a reverse effect.

Alternatively, in the case of the III-V VCSEL technology, this inversion functionality can be fully integrated with a laser matrix for example. Future work would be dedicated to dynamical control of the OAM sign (and charge) in a single device by electrical injection. To replace the static dielectric metamaterial layer used for the SPM here, one could for example integrate *μ*m size liquid crystal pixels on the 100 *μ*m size gain mirror for electro-optic control, a mature VCSEL technology. This would allow to modulate the OAM at very high frequencies in next generation modulator for quantum telecommunications.

## Materials and Methods

### Design and fabrication of the vortex laser

The 1/2-VCSEL structure was grown by MOCVD. It is composed of an epitaxial high- reflectivity (99.9%) bottom AlAs/GaAs Bragg mirror (27.5 pairs), and a GaAs active layer of 13*λ*/2 thick containing 12 strain-balanced InGaAs/GaAsP QW’s emitting at 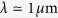. Each group of two QWs is placed at an antinode of the optical standing-wave, following a non-uniform 222020200200 longitudinal distribution (from air to Bragg) ensuring uniform QW’s carrier excitation. This ensures a low threshold carrier density and homogeneous gain broadening (with modal gain 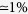 and bandwidth ~10–20 *nm*) as needed for single longitudinal mode operation^36^,^37^. We optically pumped the gain structure in the QW barriers close to Brewster incidence angle, allowing compact short millimeter-long cavity. We use 300 mW a low noise single mode 800 nm commercial diode, focused with a pair of aspheric lenses on a *w*_*p*_ = 45 *μm* spot radius.

Next to fabricate the 2D sub-*λ* grating a *λ*/8 thick *Si*_3_*N*_4_ layer was deposited on the gain mirror by ion-beam-assisted electron- beam vacuum evaporation. Then a polymethyl methacrylate (PMMA) resist was spin-coated on the wafer and patterned by electron beam lithography (Vistec EBPG 5000 at 100 kV) with 1.25 nm of resolution. After PMMA development in methylisobutylketone (MBIK) solution, the 2D grating holes are transferred to the SiN layer by Reactive Ion Etching (RIE), and at the end the PMMA is removed. Alternatively, a 10 nm thick chromium layer was deposited on the SiN layer to act as a loss mask. The same technological process was used to obtain the desired pattern. Figure 3(b1 of the paper show optical microscope images of the integrated sub-*λ* grating, we can clearly see the two fabricated phase functions.

We realized various spiral phase elements with internal diameter ranging from 6 microns to 10 microns and external radius of 20 microns for the two handedness. These values are chosen according to intensity profile of targeted *LG*_0*m*_ modes into the cavity, this is fully governed by Gaussian beam optics^34^.

The passive optical cavity is a high finesse stable plano-concave resonator of 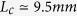. The 1/2 VCSEL integrates a flat HR mirror, and a concave output coupler (*T* = 1%) of radius of curvature *R*_*c*_ = 10 *mm* is used to close the cavity. The minimum waist for the Gaussian component of the beam occurs at the plan gain mirror and can be determined from the complex beam parameter 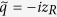. In order to lift the degeneracy of modes with different quantum number *q, p*, |*l*| the cavity length is chosen to be inside the stability region 0 < *L*_*c*_ < *R*_*c*_, far from stability edge. Once the beam parameter 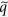 is calculated, one can write the field distribution of the complete set of LG modes basis using Eq. 1 (in the paper), and choose the suitable mask size to select the wanted mode as in the example shown in Fig. 1(c2). The typical fundamental beam waist is *w*_0_ ~ 25 *μm* here.

### Simulation of the cavity eigenstates and mode competition dynamics

To validate the method developed in this work, we used numerical simulation of the perturbed cavity eigenstates, using an approach similar to the first order perturbation theory in quantum mechanics, i.e: first we calculated eigenvalues and the corresponding eigenmodes of the cold cavity, incorporating the loss mask element centred on its axis to select the *LG*_0*l*_ doughnut mode. For this we used an in-house 2D cavity eigenmode solver based on matrix method with Huygens kernel formulation^40^. Then, once the modes calculated, we re-inject the degenerate left-handed and the right-handed helically-phased beams into the same cavity but this time containing the helical SPM and using the Fresnel diffraction based Fox-Li^41^ iterative method to study the evolution of each one to the steady-state. one can easily notice the azimuthal symmetry breakdown and degeneracy lift: indeed we see that when the helical wavefront and SPM have the same handedness, the mode conserves its homogeneous intensity distribution with an integer charge *l* (see *LG*_0,+1_ in Fig. 4(b) inset), whereas when the helical wavefront and the SPM have opposite handedness the mode is perturbed and shows significant azimuthal modulation of the intensity distribution with slightly non-integer OAM charge (see *LG*_0−1_ in Fig. 4(b) inset).

In a second step, the non-linear laser dynamics of these two - degenerate or not - modes are modeled using the spatio-temporal semi-classical Maxwell-Bloch equations^34^, in the rotating wave approximation, for a class-A laser dynamics free of relaxation oscillations (electron lifetime *A*^−1^ ≪ *γ*^−1^ photon lifetime). Population inversion is at the steady state and can be eliminated adiabatically. The QW gain, of thickness is *L*_*g*_ ≪ *λ*, is located on one end mirror. We take into account of the SPM effect, self- and cross-saturation due to azimuthal SHB in QWs, as well as spontaneous emission noise (Langevin forces *F*_*E*_) and weak back scattering 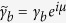 (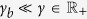; *μ* the backscattering phase). The set of dynamical equations for the slowly varying envelopes *E*_1,2_(*t*) of the E-field read:


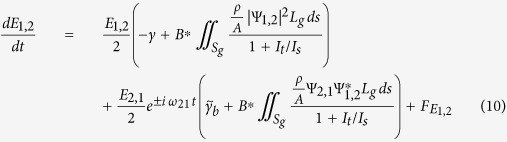


where *B*^∗^ = *B*(1 + *iα*), *B* is the Einstein coefficient for stimulated emission, *α* is the phase-amplitude coupling factor in QW (Henri factor), *ρ*(*r, θ*) is the pump distribution in QWs, *ω*_21_ is the vortex mode angular frequency difference, 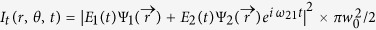 is the total photon number distribution in the QW plane, and *I*_*s*_ is the saturation photon number for *LG*_00_ mode, it is calculated for our gain chips as *I*_*s*_ = *A*/*B*^37^. We simulated the laser build up seeded by spontaneous emission noise up to the steady state, after a characteristic time of 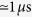 at twice threshold. The mean values of laser mode intensities and frequencies were extracted. The Table 1 below gives the laser parameters.

### Physical study of the eigenstate purity: optical beating in the RF intensity spectrum

To quantify the optical intensity suppression ratio *I*_−_/*I*_+_ of counter-rotating OAM, we measured the normalized beating power 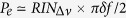 (*δf* the FWHM of a Lorentzian line-shape) between the two vortex modes at Δ*ν* in the laser RF power spectral density^34^. For this purpose we mixed the two counter-rotating vortex fields on a photo-detector and integrate spatially over a fraction *S*/3 of the mode surface (to break orthogonality). The suppression ratio *I*_−_/*I*_+_ reads (*I*_+_ for the strong mode):


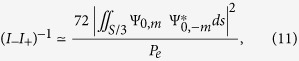


with the assumption *I*_+_ ≫ *I*_−_. It must be point out that this counter-rotating mode suppression ratio will be reduced when Δ*ν*/*f*_*c*_ is not large enough, due to a residual coherent mode coupling (due to cross-coupling terms in the right hand side of in Eq. 10).

This high resolution and sensitivity RF method is a very sensitive tool to analyse all the spectrum of existing cavity eigenstates above shot noise (polarization, transverse and longitudinal modes)^34^.

## Additional Information

**How to cite this article**: Seghilani, M. S. *et al*. Vortex Laser based on III-V semiconductor metasurface: direct generation of coherent Laguerre-Gauss modes carrying controlled orbital angular momentum. *Sci. Rep.* **6**, 38156; doi: 10.1038/srep38156 (2016).

**Publisher's note:** Springer Nature remains neutral with regard to jurisdictional claims in published maps and institutional affiliations.

## Figures and Tables

**Table 1:** Physical laser parameters used for simulation of the vortex VECSEL dynamics, w/o Spiral Phase Metasurface.

Parameters	Value	Units	Description
*γ* = 2*πf*_*cc*_	210^8^	*s*^−1^	Photon decay rate
*B*	0.7	*s*^−1^	Einstein coefficient for stimulated emission
*A*	510^8^	*s*^−1^	Carrier relaxation rate
*α*	3		Phase-amplitude coupling factor
*ω*_21_	0 or 1.2510^8^	*s*^−1^	Frequency separation of counter-rotating vortex mode
*L*_*g*_	12 × 8	*nm*	Gain thickness
*L*_*c*_	9.5	*mm*	Optical cavity length
*w*_0_	25	*μm*	*LG*00 fundamental beam waist
*γ*_*b*_	5.10^−6^*γ*	*s*^−1^	Backscattering amplitude
*μ*	*π*	*rad*	Backscattering phase
*ρ*(*r*,*θ*)	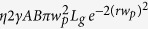	*s*^−1^*m*^−3^	Pump distribution in the QWs
*η*	1 to 2		Pump rate normalized to threshold
*w*_*p*_	45	*μm*	Pump spot radius @1/*e*^2^
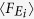	0	*s*^−1^	Random Langevin forces for the electric field of *i*^th^ mode (mean value)
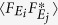	*ξγδ*(*t* − *t*′)*δ*_*ij*_	*s*^−2^	Random Langevin force variance
*ξ*	1.2		Spontaneous emission to stimulated emission ratio (reduced to one photon)
